# Design and experimental research of a novel deep-sea self-sustaining profiling float for observing the northeast off the Luzon Island

**DOI:** 10.1038/s41598-022-23208-7

**Published:** 2022-11-07

**Authors:** Qiang Wang, Zurong Qiu, Shaobo Yang, Hongyu Li, Xingfei Li

**Affiliations:** 1 College of Information and Control Engineering, Qingdao University of Technology, Qingdao, 266525 China; 2 State Key Laboratory of Precision Measuring Technology and Instruments, Tianjin University, Tianjin, 300072 China; 3 College of Ocean Science and Engineer, Shandong University of Science and Technology, Qingdao, 266590 China; 4 Qingdao National Laboratory for Marine Science and Technology, Qingdao, 266003 China

**Keywords:** Physical oceanography, Mechanical engineering

## Abstract

To understand the physical ocean laws of ocean circulation in the deep ocean below 2000 m, a profiling float named FUXING is presented to meet the deep-ocean observation requirements at a depth of 4000 m. First, to meet the low energy consumption and buoyancy regulation stability of the profiling float, the low–power buoyancy adjustment process of FUXING is effectively solved by introducing the external seawater pressure as the driving force. Then, to reduce the energy consumption of the single profile for the profiling float, the optimization of the oil draining adjustment mode in the floating process is studied. Simultaneously, a buoyancy-driven system characterization test was performed to examine the buoyancy adjustment of FUXING. When the frequency of oil draining is 15 times, the total energy consumption of FUXING is the lowest. Finally, FUXING was deployed in the northeast off the Luzon Island to validate the feasibility and reliability. The at-sea experiments indicated that the optimized oil draining adjustment mode can reduce the total energy consumption in the floating process by more than 20%. The profile data showed that the outer sea water gradually mixes with the South China Sea water after passing through the northeast off the Luzon Island.

## Introduction

In recent decades, the observation of the deep ocean layer between 2000 and 4000 m isobaths^1,2^, which would help improve estimates of the ocean heat, freshwater content, and sea level rise, has become a research hotspot^1,2^. To meet the needs of thermal content research in deep sea water, it is necessary to extend regular 2000 m profiles to deeper depths. To develop and utilize deep oceans, deep-sea observation technology is required to provide technical support. The deep-sea self-sustaining profiling float has become an important tool for accomplishing oceanic reconnaissance and different deep-sea missions, for the sustainable development of the emerging ocean industry. This has provided tremendous economic and societal benefits^3,4^.

**Figure 1:**
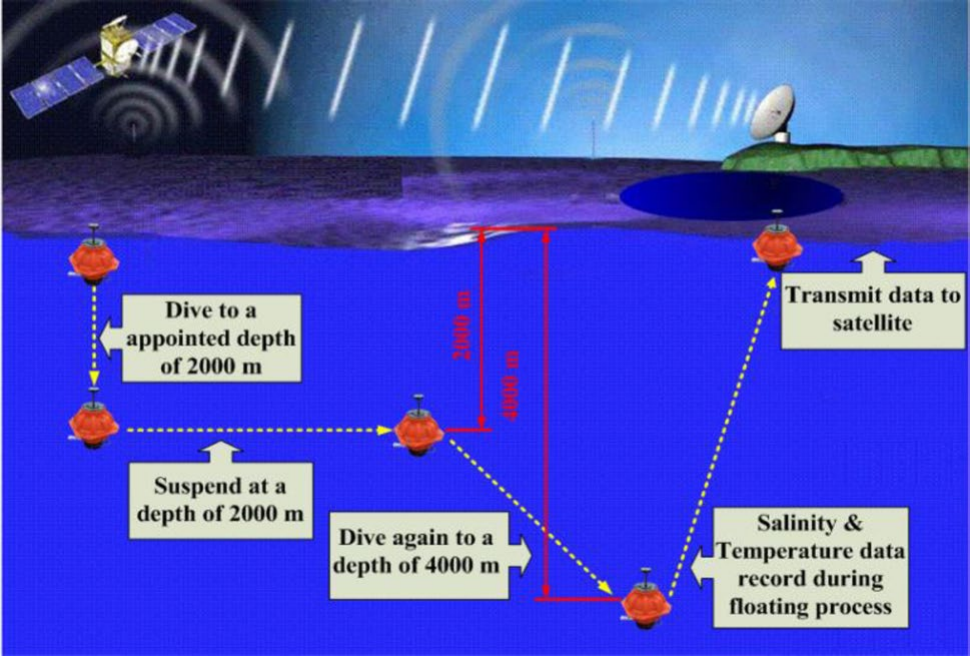
Schematic diagram of a profiling float operation cycle profile measurement.

The deep-sea self-sustaining profiling float is widely applied to the ARGO project^5^, and is called a profiling float or Argo float, which dives and ascends by altering its buoyancy. As a universal platform for ocean monitoring instruments, the profiling float can take a variety of hydrological observation sensors required by the scientific research along with it, such as temperature, salinity, depth, pressure and other sensors. It can cycle vertically and send the collected ocean hydrological data underwater to the receiver on the research vessel by iridium communication. The data is transferred toward stations on land, as shown in Fig. 1.

**Figure 2:**
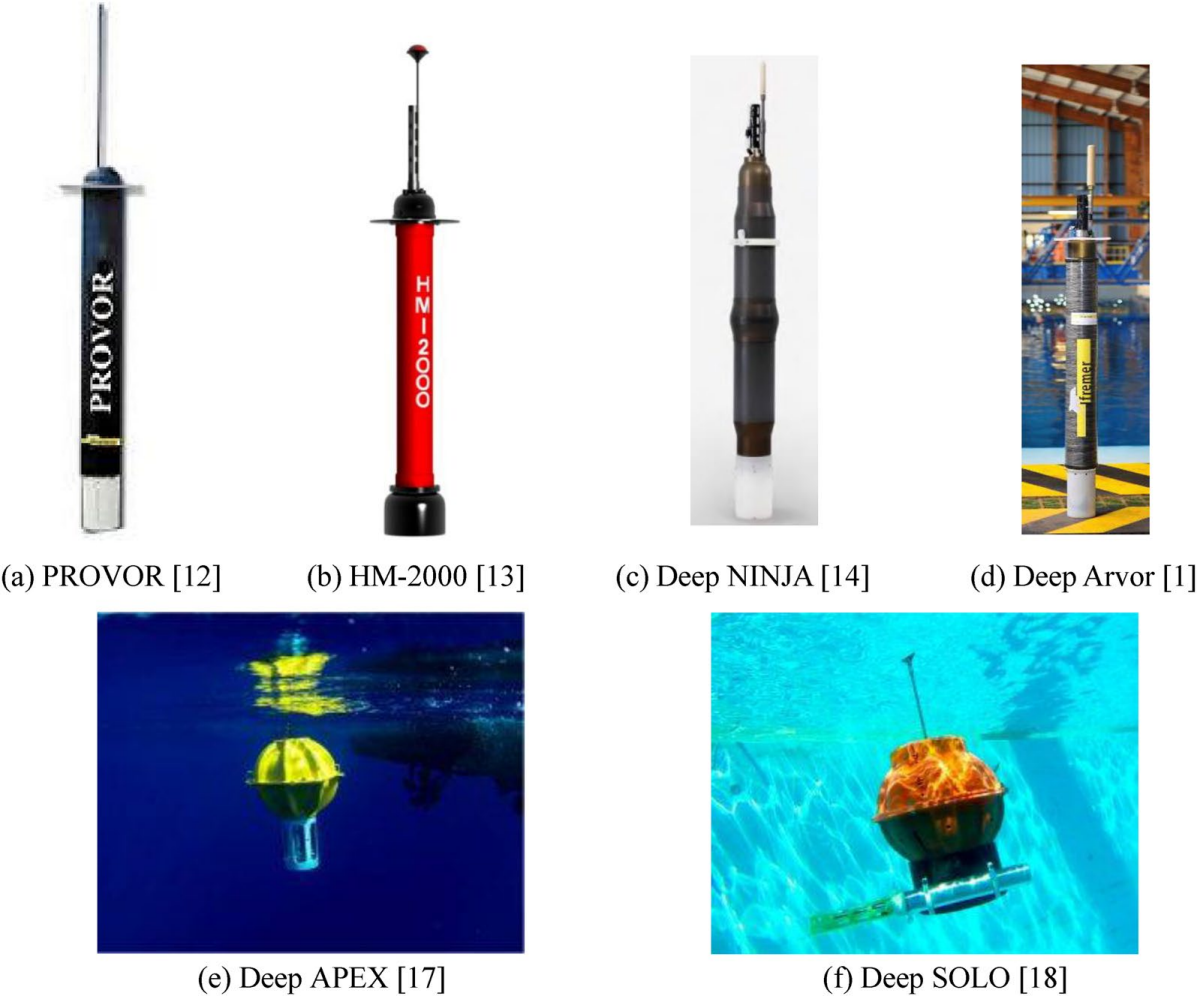
The appearance drawing of major profiling float system prototype.

A significant number of profiling floats have been developed to overcome engineering and scientific challenges caused by unpredictable underwater environments. The deep-sea self-sustaining profiling float, as a sophisticated artificial intelligence integration system, is a highly autonomous, self-learning unmanned platform that adapts to the volatile surroundings in complex ocean environments. Research on profiling float has become a research hotspot. Reviewing the development process of the intelligent float around the world, it has evolved from a neutral float proposed by Swollow to a currently utilized intelligent float^6^. Simultaneously, Stommel et al. proposed the concept of a neutral buoy to directly measure deep ocean currents^7^. For the neutral buoy^8^, because the data reception was performed by the shipboard acoustic equipment, both the drift range and the deployment depth were limited by the receiving range of the acoustic signal. The SOFAR (sound fixing and ranging) buoy developed by Rossby and Webb in 1970 had a lifespan of several years, and could be tracked over a wide range. However, the SOFAR buoy was bulky and huge, which brought significant inconvenience to the layout of equipment^7–9^. To improve the means of dumping heavy loads into the ocean in the ascending process, Davis and Webb launched the development of the autonomous Lagrangian circulation explorer (ALACE) in 1992^10,11^. The mature and successful application of alace buoy technology has enabled the United States, France, China and other countries to produce their own types of profile buoys for marine hydrological observation at a depth of 0~2000 m. Among the profiling floats currently operating in the global ocean real-time observation network, PROVOR^12^, ARVOR^1^, COPEX, and HM-2000^13^ profiling floats have been deployed in various sea areas worldwide for several years. At present, several major profiling floats have gradually been the main carrier to work under the deep-sea, such as Deep NINJA^14^, Deep Arvor^1^, Deep SOLO^18^ and Deep APEX^5^. Deep NINJA is mainly suitable for observing special environmental sea areas such as the tropical and high-latitude ice-covered seas. However, when Deep NINJA works in a sea ice environment for a long time, its buoyancy-driven system is prone to failure, resulting in a high loss probability of the profiling float. The self-ballast and lightweight characteristics of the ARVOR are maintained for the Deep Arvor. However, when the depth of Deep Arvor is greater than 2000 m, the preliminary stop challenge of the data transmission occurs owing to the deviation of the salinity value in the range of 0.01~0.02. Two types of deep floats with a spherical glass hull (Deep SOLO and Deep APEX) have been available since around 2015, which is described in the report of Zilberman and Maze (2015)^5^. But, the circuit hardware designed by Deep APEX was unstable and needs to be further optimized^17^. Compared with the hydrological data collected by SBE 9plus CTD sensor on board, the salinity profile data collected by Deep SOLO in the first 8 cycles had a certain salinity deviation (0.01~0.02 PSU pss-78)^18^. Furthermore, the Deep Arvor and Deep SOLO only met the specification of 31~32 cycles at 4000 m depth with CTD measurements. The drift at the parking depth interval was a bias of ± 50 m and ± 80 m for the Deep Arvor and Deep SOLO^16,18^. Data collection accuracy and profile work stability were the same for Deep APEX and Deep SOLO, as were Deep Arvor and Deep NINJA^15–18^. Thus, the data collection accuracy and profile operation stability of Deep Arvor and Deep SOLO need to be optimized. The major profiling float prototypes are illustrated in Fig. 2.

A few scholars have conducted extensive research on the buoyancy-driven system for the deep-sea self-sustaining profiling float. Roemmich et al.^15^ utilized a hybrid mechanism of a singlestroke to design the buoyancy-driven system for the Deep NINJA. This engine is suitable for a small vehicle operating in the deep ocean. However, the engine must pull back its piston to replenish the hydraulic oil supply after ejecting the oil. To effectively improve the buoyancy-driven engine technology of the Deep NINJA by extending its capacity to higher pressures, Le Reste et al.^16^ proposed a combination of a multistroke pump and a valve to transfer oil between an external bladder. For the designed variable buoyancy engin, A high-efficiency electric motor was chosen to optimize the energy budget. However, during immersion, the hydraulic flow rate of the valve is very pressure dependent in the design scheme. Petzrick et al.^17^ proposed an hydraulic buoyancy engine to realize the buoyancy-driven effect of the profiling float system. The design scheme mainly involved altering the effective volume utilized for the buoyancy adjustment using a direct current (DC) motor-driven pump to pump oil from the reservoir to the oil bladders. However, atmospheric pressure may be sufficient to overcome the force exerted by the spring on the rolling diaphragm to cause oil to flow from the oilbladders into the oil reservoir. The spring in the oil reservoir will reduce the retraction force due to the long-term use, which will prevent the piston from working properly in the oil reservoir. DAVID et al. adopted a motor-controlled high-pressure pump to let the oil flow from the polyurethane interior reservoir to the external bladder to increase buoyancy. But when the output flow of the pump suddenly increases, the flow of suction cannot meet the requirements in a short period of time, resulting in instantaneous suction and cavitation.

Although several major profiling floats have been utilized in the investigation and collection of marine environmental data, they are still in the development and testing stages. The profiling float still face several technical challenges, and stricter requirements have been proposed for their reliability. It has been developing toward multiple directions of multi-functional, great-submergence, and intelligence. However, the effective volume change of the buoyancy adjustment cannot be monitored for the buoyancy-driven mode of the profiling float during the buoyancy adjustment process^15–18^. In addition, the current buoyancy-driven system of the profiling float was designed to use a two-way gear pump to suction and discharge oil from the external oil bladder to achieve the depth positioning process of the profiling float^15–18^. More additional energy consumption would be consumed for the buoyancy-driven mode during the buoyancy adjustment process. The range of the profiling float is restricted. At present, the direct current (DC) motor-driven pump was only adopted to design the buoyancy-driven system of the profiling float^15–18^. But, when the inlet pressure of the bi-directional-gear pump is lower than the saturated vapor pressure of the hydraulic oil, the cavitation is easy to occur, thus reducing the volumetry efficiency of the plunger pump, the energy consumption of the profiling float is increased. In addition, the pressure housing of the profiling float still presently chooses a high-strength aluminum alloy cylindrical shell^15,16^. Strict requirements have been advocated for the compression resistance of the pressure housing. In this study, a Chinese profiling float named ‘FUXING’ was designed to meet the deep-ocean surveys needs at a depth of 4000 m.The pressure-proof structures of FUXING were designed in a spherical shape, and the chosen material of the spherical pressure hull was glass. The chosen material of the protective shell was polyethylene. The pressure resistance experiment was utilized to analyze the anti-pressure ability and compressibility of the FUXING hull. To increase the suction port pressure of the plunger pump and make full utilization of the spherical glass housing space, an air pump is used to provide an appropriate pressure for the internal reservoir to solve the air lock problem in the system. A combination of high-pressure plunger and air pumps was adopted to design the buoyancy-driven system. In order to ensure the buoyancy adjustment accuracy of the buoyancy-driven system, a pull line displacement sensor is used to precisely measure the piston displacement of the internal reservoir to realize the precise buoyancy regulation of FUXING. In the design process, the calculation model for the pressure change inside the spherical glass housing is provided. In order to reduce the energy consumption of FUXING operation process and improve the running time of it to a greater extent, the optimization of the oil draining adjustment mode for FUXING in the floating stage is proposed. The buoyancy regulation and continuous circulation capacity of the designed buoyancy-driven system at a depth of 0–4000 m were assessed by the hydraulic engine characterization test. Finally, the feasibility of the designed buoyancy-driven system and FUXING prototype was validated by continuous multi-profile performance tests in the northeast off the Luzon Island trials. The total energy consumption of profile work are analyzed and compared with the theoretical results. The obtained profile data also reveals the characteristics of the Tropical Water near the northeast off the Luzon Island area.

## Design of the FUXING buoyancy-driven system

### Buoyancy-driven system principle

**Figure 3:**
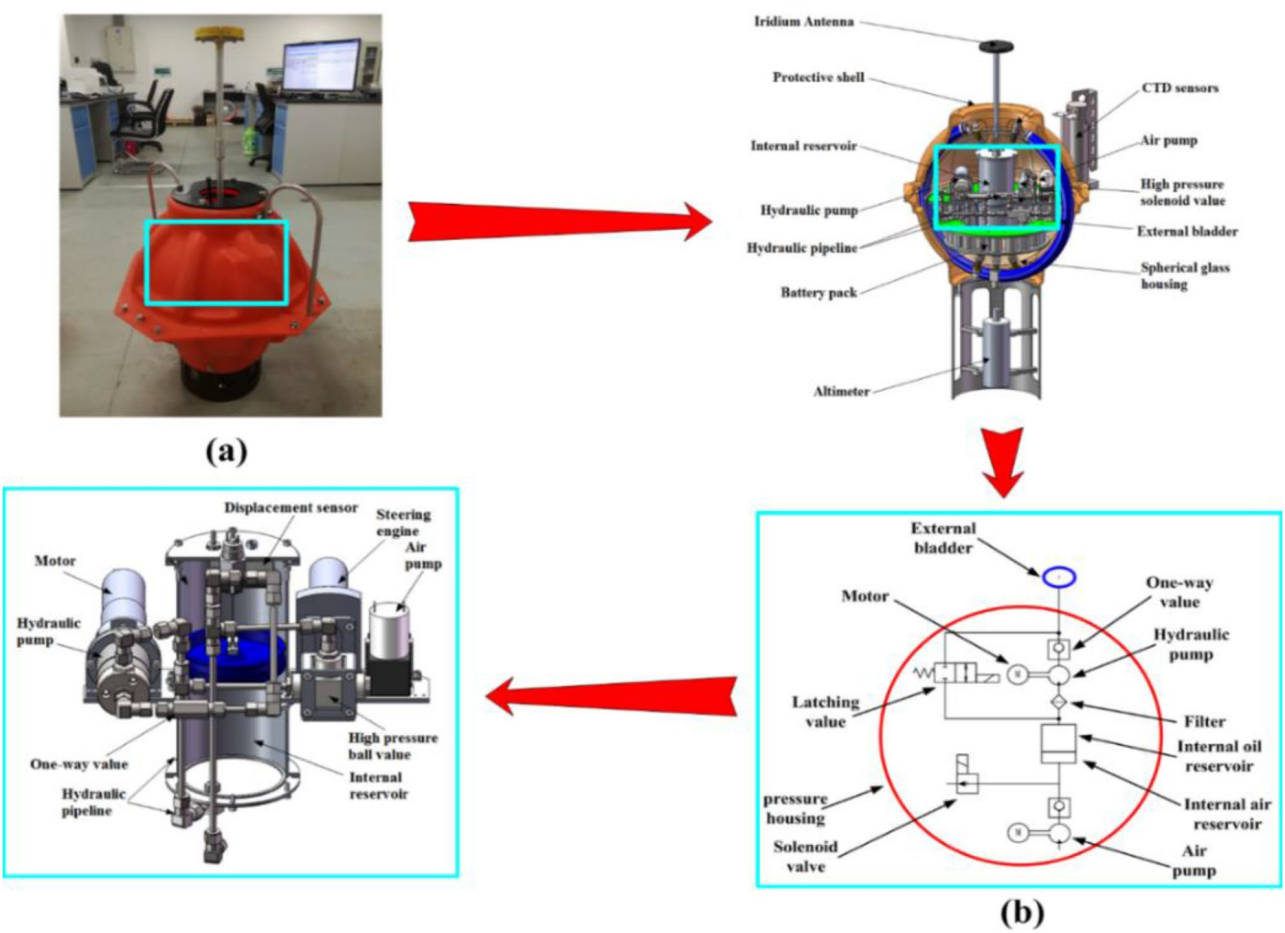
(**a**) The appearance drawing of a FUXING prototype (The blue box represents the buoyancy adjustment system position of the FUXING); (**b**) Basic schematic diagram of a buoyancy adjustment system of the FUXING.

In a complex deep-sea environment, a buoyancy-driven system can take the place of a vertical propeller and play a vital role in power-free heaving motion and autonomous drifting of the FUXING. The buoyancy regulation principles of the FUXING are mainly based on altering the displacement volumes of the FUXING to control the motion, so as to measure the water temperature, salinity, depth, and other data. The buoyancy-driven system schematic of FUXING and its appearance are illustrated in Fig. 3.^19^

When FUXING is ready to submerge, a latching value is opened to pump the hydraulic oil of the external oil bladder into the internal oil reservoir. FUXING dives to a predefined depth because the volume of the external oil bladder becomes small to decrease the buoyancy. FUXING will be suspended in seawater for the scheduled period. When FUXING ascends, hydraulic oil flows from the internal oil reservoir through a filter to a hydraulic pump, which is driven by the motor. The single-stroke plunger pump is replaced by a smaller high-pressure plunger pump to provide ultrahigh pressure. A hydraulic pump can utilize the sphere space fully. Hydraulic oil is pumped into the external oil bladder to increase its buoyancy using a one-way value. The one-way value can prevent hydraulic oil from reversing backflow through the smaller high-pressure pressure plunger pump. The buoyancy of the FUXING begins to increase with the volume of the external oil bladder expanding. The FUXING will ascend when the buoyancy is greater than the gravity of FUXING. The vertical profile data of water temperature, pressure, and salinity will be collected by the CTD sensors during their ascent to the surface. When FUXING is pushed to the surface, the data can be transmitted via satellites. After the data are transmitted, the new dive will start to the next period until the battery is out of power^20^.

### Minimum volume of internal oil reservoir for buoyancy adjustment

The relevant specifications of the FUXING listed in Table 1.

**Table 1:** Relevant parameters and configuration of FUXING specifications.

Description	Value
FUXING height	0.82 m
FUXING maximum width	0.54 m
Total mass of FUXING	52.39 kg
Own volume of FUXING	0.0504m^3^
Maximum diving depth	4000 m
Methods for data transfer and positioning	Iridium communication, GPS positioning
Payload capacity	Salinity, temperature, depth, pH, ORP, SVP sensors, current meter

**Figure 4:**
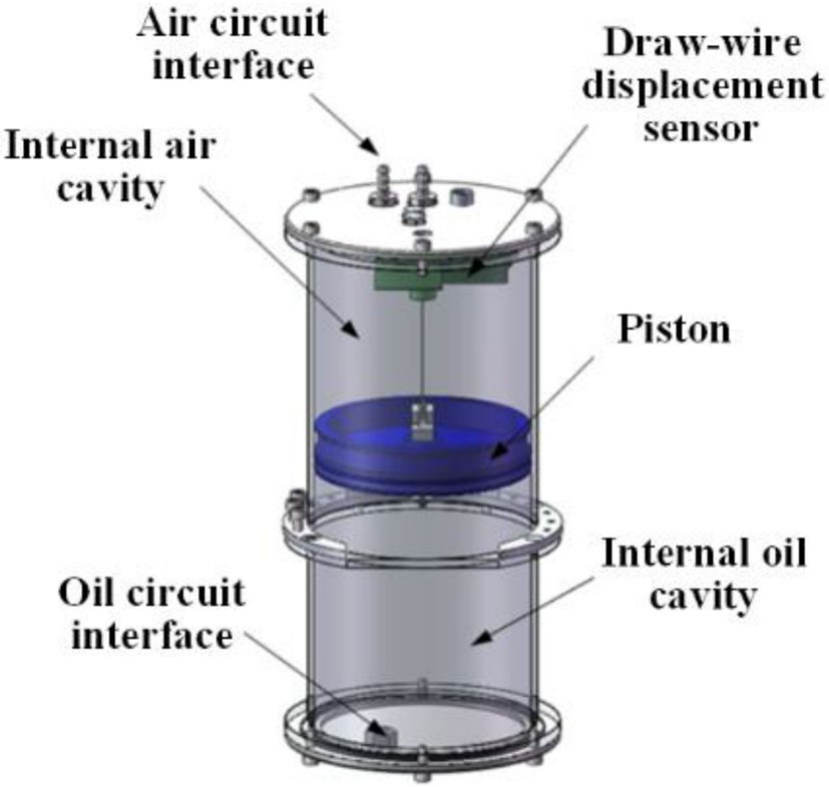
Internal structure perspective of the internal oil reservoir.

Because the total amount of hydraulic oil in the buoyancy-driven system is known, if the amount of hydraulic oil in the internal oil reservoir is determined, the amount of hydraulic oil in the external oil bladder can be calculated. Thus, the buoyancy provided by the external oil bladder to the FUXING can be accurately measured. Therefore, the design of the internal oil reservoir component plays an important role in the design process of the entire buoyancy-driven system. To realize the compact structure and good modularization of the buoyancy-driven system, to utilize less hydraulic oil to change the larger buoyancy, and the oil quantity can be accurately adjusted; a piston-type internal oil reservoir assembly structure is designed in this study. Figure 4 illustrates the structural diagram of the internal oil reservoir, which comprises a piston, internal oil and air cavities, air and oil circuit interfaces, and a draw-wire displacement sensor. A high-precision draw-wire displacement sensor was installed in the inner oil cavity. According to the piston area *S*, and displacement ∆*h* of the displacement sensor, the oil quantity change in the internal oil cavity can be calculated as *Q* = *S* × ∆*h*. Therefore, the buoyancy adjustment can be indirectly calculated based on the amount of oil change.

Based on the forces acting on the FUXING, combined with the FUXING hull deformation and the variation law of seawater density, the limit idea is utilized to determine the mass of the counterbalancing weight before launching the FUXING, and the volume of internal oil reservoir required for buoyancy adjustment. The force equilibrium equation of the FUXING is expressed as follows:

1 $$\left\{ {\begin{array}{*{20}l} {F_{a} = (M_{f} + M_{Fe} )g - \rho g(V_{f} + V_{Fe} + Q - \Delta V) - \frac{1}{2}C_{d} Av^{2} } \hfill \\ {Q = Q_{1} + Q_{2} } \hfill \\ \end{array} } \right.$$

where *F*_*a*_ is the resultant force of the FUXING under water; *M*_*f*_ is the total mass of the FUXING; *M*_*Fe*_ is the mass of the counterbalancing weight of the FUXING, *g* is the acceleration due to gravity, *ρ* is the seawater density, *V*_*f*_ is the profiling float volume at 1 atm, *V*_*Fe*_ is the drainage volume of the counter-balancing weight inside the FUXING, *∆V* is the profiling float hull deformation caused by the underwater environment pressure and temperature, *C*_*d*_ is the drag coefficient (*C*_*d*_ = 0.7), *A* is the projected area of the FUXING hull (*A* = 0.301 m^2^), *v* is the mean vertical velocity of the FUXING in both the ascending and diving processes (*v* = 0.1 m/s) and *Q* is the hydraulic oil volume of the external oil bladder. For the convenience of analysis, the regulating oil volume of the external oil bladder is divided into two parts: one part is the neutral buoyancy regulating oil volume *Q*_1_ related to gravity balance, and the other is the non-neutral buoyancy regulating oil volume *Q*_2_ related to water resistance balance.

According to Eq. (1), the force equilibrium equation of the FUXING can be obtained as follows:

2 $$F_{a} = (M_{f} + M_{Fe} )g - \rho g(V_{f} + Q_{1} - \Delta V) - \rho gQ_{2} - \frac{1}{2}\rho C_{d} Av^{2}$$

When the FUXING reaches its neutral buoyancy at depths of 10 m and 4000 m, the force equilibrium equation for the static condition is obtained as:

3 $$\left\{ {\begin{array}{*{20}l} {\rho_{10} g(V_{f} + V_{Fe} + Q_{1} ) - (M_{f} + M_{Fe} )g = 0} \hfill \\ {\rho_{4000} g(V_{f} + V_{Fe} - \Delta V_{4000} ) - (M_{f} + M_{Fe} )g = 0} \hfill \\ \end{array} } \right.$$

where *ρ*_10_ and *ρ*_4000_ are the seawater densities at depths of 10 m and 4000 m, respectively. *∆V*_4000_ is the FUXING hull deformation caused by the underwater environment pressure and temperature at a depth of 4000 m. It can be derived from Eq. (3) that *Q*_1_ is mainly determined by the regulating oil volume of the external oil bladder caused by the seawater density variation and the profiling float hull deformation.

**Figure 5:**
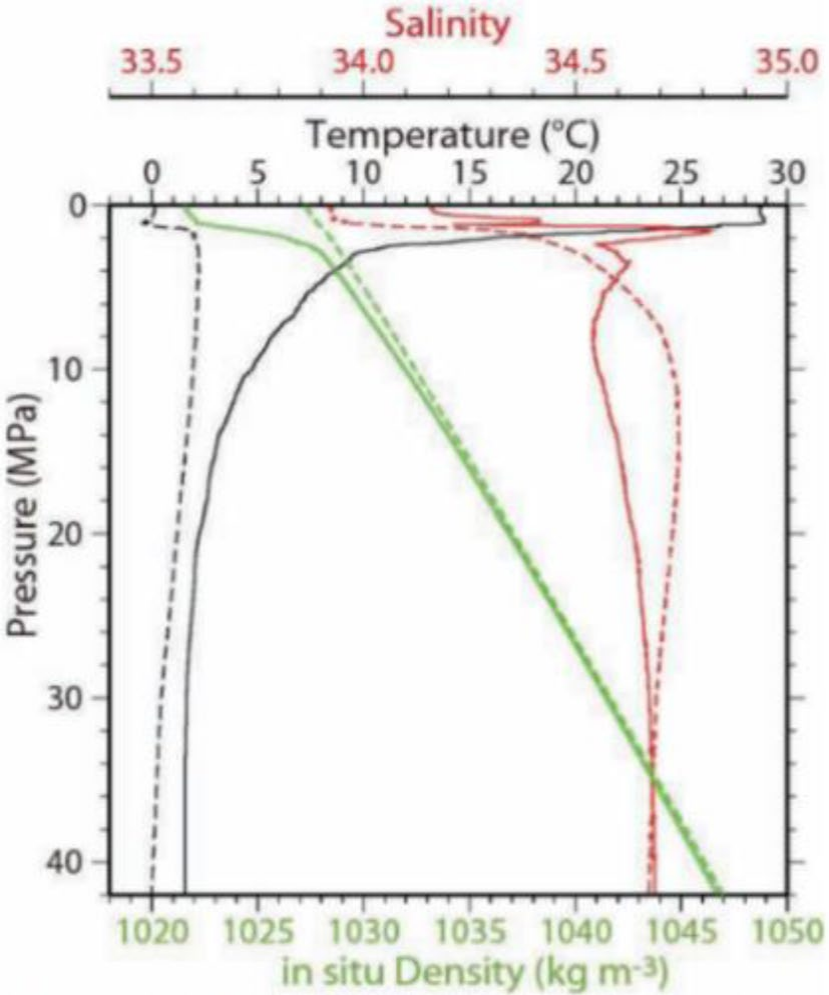
The typical hydrological data in the Western Pacific and North Atlantic by the NINJA float^21^.

**Figure 6:**
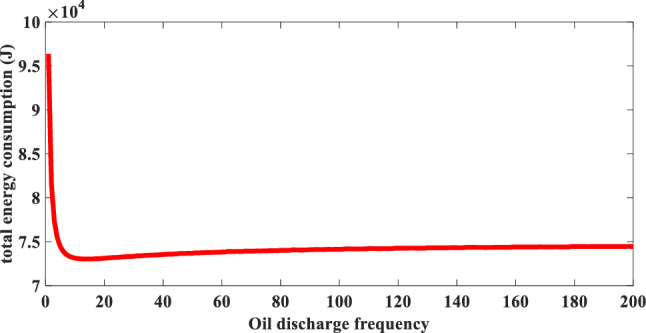
The relationship between different oil draining frequency and total energy consumption of FUXING.

Figure 5^21^ illustrates the typical hydrological data cited by Japanese scholars in the technical demonstration related to the development of the NINJA float at the 2012 ISOPE conference, corresponding to the hottest and coldest, maximum, and minimum surface densities, respectively. It can be utilized as a reference for studying the maximum variation in sea water density. In Fig. 6, the solid and dotted lines represent the hydrological data of the Western Pacific and the North Atlantic, respectively. The limit variation range of seawater density at a water depth of 10 m to 4000 m is 1.021 × 10^3^ kg/m^3^ to 1.047 × 10^3^ kg/m^3^.

According to the calculation equation of the FUXING hull deformation caused by the underwater environment pressure and temperature^19^, the FUXING hull deformation is approximately 800 ml at a depth of 4000 m. If the total mass of the FUXING is 46 kg, the counterbalancing weight is the stainless-steel weight combination of different weights, and the density of stainless steel is 7980 kg/m^3^. The counterbalancing weight of the FUXING system is calculated from Eq. (3) as 5.87 kg.

Considering that the total displacement of the designed FUXING system should not exceed 60 kg, it can be calculated from Eq. (3) that the regulating oil volume of the external oil bladder for the buoyancy adjustment is approximately 632 ml. In addition, considering the compressibility of hydraulic oil under high pressure at a water depth of 4000 m, the hydraulic oil compressibility of the external oil bladder is 1.5 × 109 Pa. The maximal adjustable oil compression under a pressure of 40 MPa is 20 ml. According to the aforementioned calculation process, the neutral buoyancy regulating oil volume *Q*_1_ related to gravity balance is 652 ml.

The non-neutral buoyancy regulating oil volume *Q*_2_ related to the water resistance balance is mainly utilized to regulate the upward and downward movements of the FUXING. Because the mean vertical velocity of the FUXING is approximately 0.1 m/s in both the ascending and diving processes, it can be approximated as a uniform motion. Equation (2) can be obtained as follows:

4 $$- \rho gQ_{2} - \frac{1}{2}\rho C_{d} Av^{2} = 0$$

According to (2), the regulating oil volume of the external oil bladder that needs to be adjusted to stabilize the mean vertical velocity of the FUXING at 0.1 m/s is approximately 108 ml. Considering that the oil loss in the high-pressure pipeline is approximately 140 ml, the calculation results indicate that the minimum volume of the required internal oil reservoir for buoyancy adjustment is approximately 900 ml.

### Analysis and calculation of air pressure variation in spherical glass housing

The end face seal was adopted between the upper and lower hemispherical shells of the spherical glass housing. It is necessary to extract a vacuum to complete the sealing of the spherical glass housing. Currently, a negative pressure state was formed in the spherical glass housing. In the working process of the buoyancy-driven system, the smaller high-pressure plunger pump has the problem of insufficient oil suction capacity, which leads to the vacuum environment, high-speed oil suction is easy to produce a vacuum chamber at the oil inlet end. That is, when the high-pressure plunger pump outputs the hydraulic oil in the internal oil reservoir placed in the negative pressure environment to the external oil bladder, it is easy to separate small vacuum bubbles at the oil inlet of the high-pressure plunger pump, resulting in air lock, which makes it difficult to output the hydraulic oil to the external oil bladder.

To solve the air lock problem and enhance the oil absorption capacity of the plunger pump, an air pump is required to pressurize the inner oil reservoir, which inevitably leads to air pressure changes inside the spherical glass housing. To ensure that the high-pressure plunger pump has a normal suction pressure during the ascending and diving processes of the FUXING, it is necessary to analyze the pressure change inside the spherical glass housing during this process.

#### Calculation of internal vacuum degree in the spherical glass housing

The vacuum degree is a measure of the pumped air, which means that the actual value of the system pressure is lower than the atmospheric pressure. That is, vacuum (Z) = atmospheric pressure—absolute pressure, absolute pressure = atmospheric pressure + gauge pressure (− vacuum)^22,23^. Before calculating the vacuum degree inside the spherical glass housing, it was necessary to determine the residual gas inside the spherical glass housing. The volume occupied by the buoyancy drive system, control system, and battery pack in the spherical glass housing was removed, and the remaining volume (V remaining volume) was the volume of residual gas. Table 2 presents the volume of each component required to calculate the remaining gas.

**Table 2:** Volume value occupied by each component inside and outside of spherical glass housing.

Parameter	Value (m^3^)	Description
*V*_2_	0.003718 m^3^	Volume occupied by the battery pack assembly
*V*_3_	0.001554 m^3^	Volume occupied by a hydraulic system
*V*_4_	0.0009 m^3^	Internal volume occupied by the hydraulic oil
*V*_5_	0.0003 m^3^	Upper space volume of the internal oil capsule piston
*V*_6_	0.0001 m^3^	Volume required for the control system
*V*_7_	0.00055 m^3^	Required volume of the external air bladder

Given that the internal cavity diameter of the spherical glass housing is 405 mm, the maximum volume space contained in the spherical glass housing is *V*_1_ = 0.034783 m^3^, and the remaining gas volume inside the spherical glass housing (*V*_remaining volume_) is calculated as follows:

5 $$V_{remaining \, volume} = V_{1} - V_{2} - V_{3} - V_{6}$$

The minimum volume of the internal oil reservoir is *V*_4_ = 900 ml, the volume of the designed air bladder is *V*_7_ = 550 ml, and the rated pressure safety factor of the external supplementary internal air chamber and the air bladder is 1.2. Then, the actual vacuum degree of the spherical glass housing (*Z′*) is calculated as follows:

6 $$Z^{^{\prime}} = ((V_{remaining \, volume} \times 0.5 + V_{4} + V_{7} \times 1.2)/V_{remaining \, volume} ) \times 100\%$$

Using the aforementioned calculation, it can be observed that the internal air pressure value that guarantees the sealing of the spherical glass housing is approximately 0.56 bar.

#### Analysis and calculation of pressure change in spherical glass housing when diving and floating

It is known that the sealing of the spherical glass housing for the internal initial air pressure is *P*_1_ = 0.56 bar. According to the selection of the high-pressure plunger and air pumps, the pressure of the external supplementary air bladder is *P*_2_ = 1.2 bar, and ensuring the oil suction pressure range of the high-pressure piston pump for the normal operation of the hydraulic system is *P*_3_ = 0.8–1.5 bar.

When the minimum suction pressure of the high-pressure plunger pump (*P*_3_) is 0.8 bar, the pre-pressure on the piston of the internal oil reservoir (*P*_4_) is:

7 $$P_{4} = \frac{{P_{3} (V_{4} + V_{5} )}}{{V_{5} }}$$

When the minimum suction pressure of the high-pressure plunger pump (*P*′_3_) is 1.5 bar, the pre-pressure on the piston of the internal oil reservoir (*P*′_4_) is calculated as follows:

8 $$P^{\prime}_{4} = \frac{{P^{\prime}_{3} (V_{4} + V_{5} )}}{{V_{5} }}$$

The internal gas pressure when the spherical glass housing is submerged (*P*_0_) is calculated as follows:

9 $$P_{0} = \frac{{(P_{1} V_{remaining \, volume} + P_{2} V_{7} )}}{{(V_{remaining \, volume} - V_{4} )}}$$

Whether the temperature difference between the temperature at a depth of 4000 m and the sea surface temperature affects the internal pressure change of the spherical glass housing seal during the floating process will be further discussed below.

1. The influence of the temperature difference between the temperature at a depth of 4000 m and the sea surface temperature is not considered.
  When the minimum oil suction pressure of the high-pressure piston pump (*P*_3_) is 0.8 bar, the internal gas pressure as the spherical glass housing ascending (*P*_n_) is calculated as follows:
  10 $$P_{n} = P_{0} - \frac{{P_{4} V_{5} }}{{V_{{_{remaining \, volume} }} - V_{4} }}$$
  When the minimum oil suction pressure of the high-pressure piston pump (*P*′_3_) is 1.5 bar, the internal gas pressure as the spherical glass housing ascending (*P*′_n_) is calculated as follows:
  11 $$P^{\prime}_{n} = P_{0} - \frac{{P^{\prime}_{4} V_{5} }}{{V_{{_{remaining \, volume} }} - V_{4} }}$$
2. Considering the influence of the temperature difference between the temperature at a depth of 4000 m and the sea surface temperature, it is assumed that the sea surface temperature is *T*_0_ = 29 °C, and the temperature at a water depth of 4000 m is *T*_n_ = 1.6 °C.
  When the minimum oil suction pressure of the high-pressure piston pump (*P*_3_) is 0.8 bar, the internal gas pressure obtained as the spherical glass housing ascends (*P*_*t*n_) is calculated as:
  12 $$P_{tn} = \frac{{(T_{n} P_{0} (V_{remaining \, volume} - V_{{4}} ) - T_{0} P_{4} V_{5} )}}{{T_{0} (V_{remaining \, volume} - V_{{4}} - V_{5} )}}$$
  When the minimum oil suction pressure of the high-pressure piston pump (*P*′_3_) is 1.5 bar, the internal gas pressure obtained as the spherical glass housing ascends (*P*′_*t*n_) is calculated as:
  13 $$P^{\prime}_{tn} = \frac{{(T_{n} P_{0} (V_{{_{remaining \, volume} }} - V_{{4}} ) - T_{0} P^{\prime}_{4} V_{5} )}}{{T_{0} (V_{{_{remaining \, volume} }} - V_{{4}} - V_{5} )}}$$

It can be observed from the calculation results whether the influence of the temperature difference between the temperature at a depth of 4000 m and the sea surface temperature is considered. When the oil suction pressure range of the high-pressure plunger pump is 0.8–1.5 bar, the calculation results of the internal gas pressure for the spherical glass housing ascending process meet the internal pressure requirements of the spherical glass housing seal. Furthermore, the gas pressure inside the spherical glass housing under the influence of the seawater temperature difference was lower than that without, considering the influence of the seawater temperature difference. The required parameters and numerical calculation results are presented in Table 3.

**Table 3:** Parameters and results obtained from calculating the internal pressure of spherical glass housing when the profiling float diving and floating.

Parameter	Value (bar)	Description
*P*_1_	0.56 bar	Ensure the internal pressure of the spherical glass housing seal
*P*_2_	1.2 bar	Pressure for external supplementary air bladder
*P*_3_	0.8 bar	Minimum pressure to ensure the normal operation of high pressure plunger pump
*P*′_3_	1.5 bar	Maximum pressure to ensure the normal operation of high pressure plunger pump
*P*_4_	3.2 bar	The pre-pressure on the piston of the internal oil reservoir under the minimum suction pressure of the high pressure plunger pump
*P*′_4_	6 bar	The pre-pressure on the piston of the internal oil reservoir under the maximum suction pressure of the high pressure plunger pump
*P*_0_	0.6 bar	The gas pressure inside the spherical glass housing as it is diving
*P*_n_	0.566 bar	Without considering the influence of the seawater temperature difference, the internal gas pressure when the spherical glass housing ascending under the minimum suction pressure of the high pressure plunger pump
*P*′_n_	0.537 bar	Without considering the influence of the seawater temperature difference, the internal gas pressure when the spherical glass housing ascending under the maximum suction pressure of the high pressure plunger pump
*P*_tn_	0.517 bar	Considering the influence of the seawater temperature difference, the internal gas pressure when the spherical glass housing ascending under the minimum suction pressure of the high pressure plunger pump
*P*′_tn_	0.488 bar	Considering the influence of the seawater temperature difference, the internal gas pressure when the spherical glass housing ascending under the maximum suction pressure of the high pressure plunger pump

### Analysis and optimization of energy consumption

The energy consumption of single profile motion has a decisive influence on the endurance of FUXING system. Thus, the energy budget estimation for FUXING is analyzed, and the power consumption of four parts including the buoyancy-driven system, the control system, the satellite communication system and the CTD sensor during the movement of a profile is mainly studied in this paper. According to the working voltage, current and working time of the aforementioned energy-consuming units, the energy consumption required for a single profile at a depth of 4000 m can be calculated. The specific energy consumption of each unit is shown in Table 4.

**Table 4:** Specific energy consumption of each unit.

Energy-consuming units	Energy consumption [kJ/cycle] 4000 m profile case
Buoyancy-driven system	59.57
Control system	9.6
Satellite communication system	1.32
CTD sensor	19.2
Total	89.69

In the aforementioned energy consumption units, the main energy consumption unit of FUXING is buoyancy-driven system. Thus, the key issue to reduce the energy consumption of each profile is to optimize the energy consumption of the buoyancy-driven system. According to the analysis of the working process of FUXING, it is found that the power of the floating process is much greater than that of the diving process. In the ascent process of the profiling float, the total amount of hydraulic oil of the internal oil reservoir can be transferred to the external oil bladder by driving the motor at one time. However, to make full use of the inertial motion of the profiling float in the water and achieve the purpose of energy saving, the required oil draining amount of the profiling float is completed at different frequency by intermittently starting the motor in the floating process. For the floating process, the energy consumption of the buoyancy-driven system at different frequency of the oil draining and traditional one-time oil draining oil is analyzed in this paper.

According to the selection of the high-pressure piston pump, the approximate representation of the relationship between the input torque of the high-pressure piston pump (*T*_1_) and the seawater pressure (*p*) can be determined as^24.^

14 $$T_{1} = 2.1 \times 10^{ - 2} p$$

The relationship between starting power consumption of DC motor (*W*_s_) and its load torque (*T*_2_) is expressed as^24.^

15 $$W_{S} = 666.7T_{2} + 166.7$$

The relationship between the motor stable operating power (*P*_*e*_) and its load torque (*T*_2_) is expressed as^33^

16 $$P_{e} = 333.3T_{2} + 83.3$$

In order to analyze the influence of the oil draining frequency of the buoyancy-driven system on the total energy consumption, the method for multiple quantitative oil draining of the buoyancy-driven system is explored to study the energy consumption optimization of FUXING during the floating process. If the frequency of oil draining of the buoyancy-driven system is (*n*) during the ascent of FUXING, and the minimum volume of the internal oil reservoir is 900 ml as the total oil draining, then the oil volume obtained by the current *i*-th adjustment of the external oil bladder through the buoyancy-driven system (*v*) is expressed as:

17 $$v = \frac{900(i - 1)}{n}$$

When the FUXING is in a neutral buoyancy state, the FUXING remains suspended, and the following relationship condition is satisfied:

18 $$mg = \rho (z)g[V_{f} - \Delta V + 900(i - 1)/n]$$

where*, V*_*f*_ is the FUXING volume at 1 atm; *∆V* is the FUXING hull deformation caused by the underwater environment pressure and temperature ($\Delta V = 1.844 \times 10^{ - 11} \rho gz$^19^); *m* is the total mass of FUXING; *g* is the acceleration due to gravity; *ρ* (*z*) is the seawater density at the seawater depth *z.* Due to the fitting formula of the seawater density in the South China Sea is expressed as^19.^

19 $$\, \rho {(}z{\text{) = 1028e}}^{{{4}{\text{.364}} \times {10}^{{ - 6}} z}} - 7.228{\text{e}}^{{{ - 6}{\text{.636}} \times {10}^{{ - 3}} z}}$$

From Eq. (19), the expression of the depth *z* can be calculated as follows:

20 $$z{ = } - 150.59\rho (z)\log [142.22\rho (z)]$$

According to Eqs. (17), (18) and (19), the input torque of the high-pressure plunger pump during the *i*th adjustment process (*T*_*i*_) is expressed as:

21 $$T_{i} = \frac{{2.1 \times 10^{ - 2} mngz}}{{nV_{0} - 1.844 \times 10^{ - 11} \times ({\text{1028e}}^{{{4}{\text{.364}} \times {10}^{{ - 6}} {\text{z}}}} - 7.228{\text{e}}^{{{ - 6}{\text{.636}} \times {10}^{{ - 3}} {\text{z}}}} )gz + (i - 1)V}}$$

From the single oil draining of the high-pressure plunger pump and the displacement parameters of the pump, the working time of the DC motor corresponding to different oil draining frequency (*t*) can be expressed as follows:

22 $$t = \frac{900}{{n(100/60)}} = 540/n$$

Then, according to Eqs. (15) and (16), the power consumption of each adjustment of the DC motor (*W*_*i*_) is expressed as:

23 $$W_{i} = 666.7T_{i} + 166.7 + \frac{{540(333.3T_{i} + 83.3)}}{n}$$

Thus, the total power consumption of FUXING buoyancy-driven system during the floating process (*W*) is expressed as:

24 $$W = \sum\limits_{i = 1}^{n} {W_{i} }$$

It can be found from Table 4 that the main energy consumption units of FUXING are mainly concentrated in three parts: the buoyancy-driven system, the control system and the CTD sensor. The energy consumption of the buoyancy-driven system changes with the increasing of the oil draining frequency, but the increased operating time causes the energy consumption of the control system to increase. Combining with the operating energy consumption of the control system, the CTD sensor and the total power consumption of the buoyancy-driven system during the floating process, the relationship between different oil draining frequency and the total energy consumption of FUXING was obtained through the reliability test, as shown in Fig. 6. It can be seen from Fig. 6 that the relationship between the oil drainage frequency of the buoyancy-driven system and the total energy consumption of FUXING is nonlinear. With the increasing of the oil drainage frequency of the buoyancy-driven system, the total energy consumption of FUXING first decreases and then slowly increases. When the frequency of the oil draining is one time, the total energy consumption of FUXING is 96.4 kJ, and the total energy consumption is the maximum value at this frequency. When the frequency of the oil draining is 15 times, the total energy consumption of FUXING is 73 kJ, and the total energy consumption is the lowest at this frequency. Compared with the required energy consumption value to discharge the total amount in the internal oil reservoir at one time, 24.2% energy consumption is saved.

## Laboratory tests

The reliability test of the buoyancy-driven system and the pressure-proof test of the FUXING were performed before the FUXING was deployed in the the Northeast off the Luzon Island.

### Reliability test of the buoyancy-driven system

In a complex deep-sea environment, the buoyancy-driven system can replace the vertical propeller and play a vital role in the power-free heaving motion and depth control of the FUXING. Thus, it is necessary to conduct a reliability test of the buoyancy-driven system. The flow rate and supply current of the buoyancy-driven system were assessed in this study. The energy budgets versus operating pressures were calculated. Moreover, the buoyancy-driven system was checked for any leakage or abnormal deformation under high pressure^25–28^.

**Figure 7:**
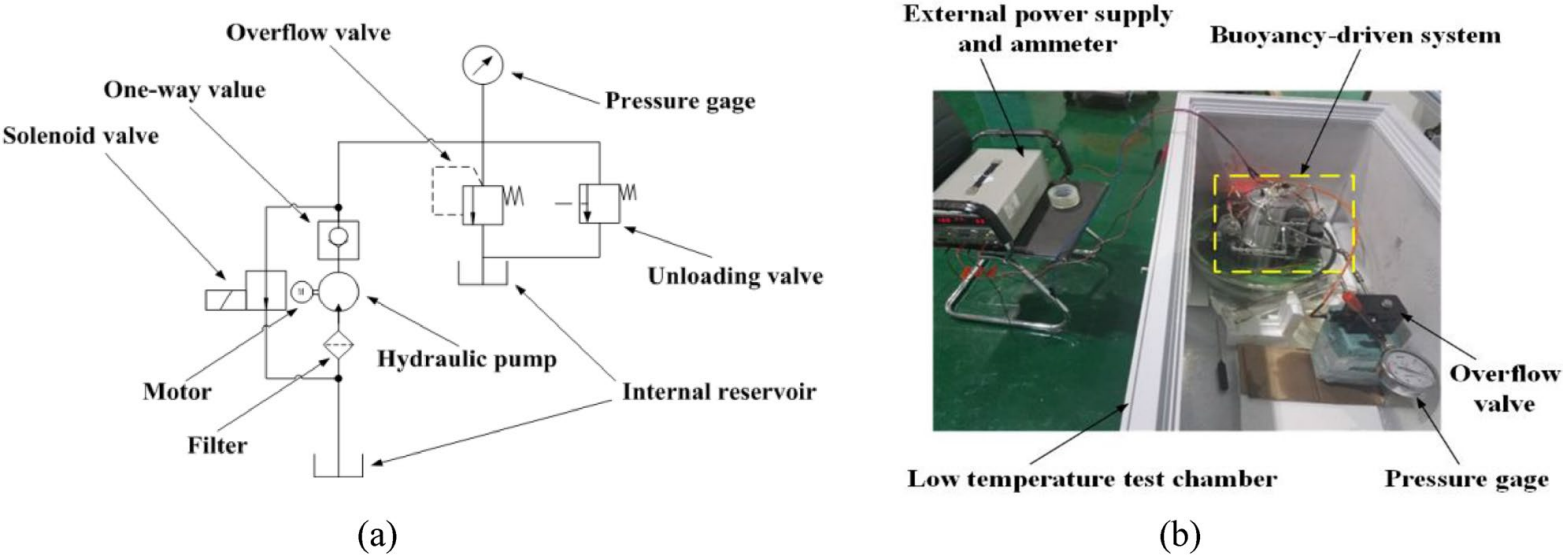
Hydraulic reliability test used for the assessment of the buoyancy-driven system efficiency: (**a**) Hydraulic reliability test used for the assessment schematic of the buoyancy-driven system; (**b**) Hydraulic reliability test photo used for the assessment schematic of the buoyancy-driven system at 2 ℃

The average temperatures of the sea surface and 4000 m in the Northeast off the Luzon Island are approximately 25 °C and 2 °C, respectively. The working state of the buoyancy-driven system could not be determined at 2 °C and 25 °C. In this study, the working state of the buoyancy-driven system at 2 °C and 25 °C was analyzed. A reliability test schematic of the buoyancy-driven system is illustrated in Fig. 7. The buoyancy-driven system was powered by an external power supply. The external bladder is removed, the installation interface of the external bladder is blocked by the tooling, and peripheral equipment such as an overflow valve, pressure gauge, unloading valve, and multimeter are connected. The control program is started to run the buoyancy-driven system according to the given debugging process. The high-pressure plunger pump was activated, and the volume of the transferred oil was measured by weighing the internal oil reservoir. The overflow pressure of the overflow valve was adjusted to 5 MPa. The entire overflow pressure range of the overflow valve was 0 MPa–40 MPa with a pressure gradient of 5 MPa. When the pressure reached the preset pressure value, the preset pressure value was maintained for 5 min. The maximum and minimum values of the multimeter versus pressure were recorded during this process.

**Figure 8:**
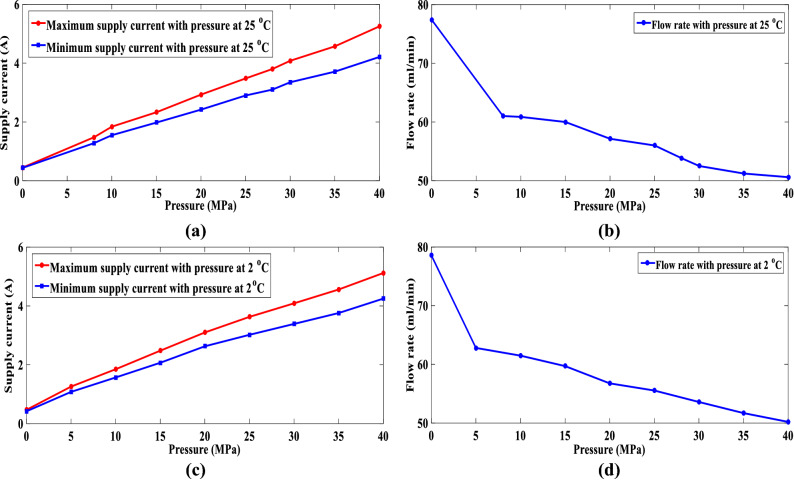
Maximum and minimum current and flow rate absorbed by the pump during oil transfers under different pressure: (**a**) Variation curve of the supply current absorbed by the pump during oil transfers with pressure at 25 °C; (**b**) Variation curve of the flow rate absorbed by the pump during oil transfers with pressure at 2 °C; (**c**) Variation curve of the supply current absorbed by the pump during oil transfers with pressure at 2 °C; (**d**) Variation curve of the flow rate absorbed by the pump during oil transfers with pressure at 2 °C.

Figure 8a,c illustrate the variation curve of the supply current under various pressure from 0 to 40 MPa at 25 °C and 2 °C, respectively. The red and blue solid lines represent the maximum and minimum supply current variation curves of the buoyancy-driven system under various pressure from 0 to 40 MPa, respectively. Figure 8b,d illustrate the variation curve of the flow rate under various pressure from 0 to 40 MPa at 25 °C and 2 °C, respectively. According to the reliability test results of the buoyancy-driven system, it can be observed that the supply current increases linearly with increasing pressure when the buoyancy-driven system runs at 2 °C or 25 °C. The flow rate of the buoyancy-driven system decreased with increasing pressure at 2 °C or 25 °C. Moreover, when the buoyancy-driven system runs at 2 °C or 25 °C the maximum supply current may not be over 5.5 A, and the flow rate may not be less than 45 ml/min. The buoyancy-driven system has no leakage or abnormal deformation under various pressures from 0 to 40 MPa at 2 °C or 25 °C. Because the tested maximum working pressure is 40 Mpa, the buoyancy-driven system has the ability to absorb and drain oil at a depth of 4000 m during the oil transfer process. The design requirements of the buoyancy-driven system were verified.

**Figure 9:**
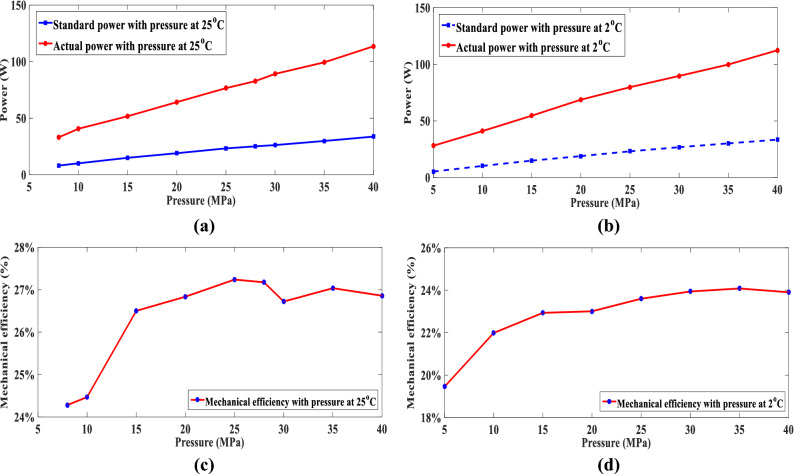
Standard power, actual power and mechanical efficiency absorbed by the pump during oil transfers under different pressure: (**a**) Variation curve of the standard power and actual power absorbed by the pump during oil transfers with pressure at 25 °C; (**b**) Variation curve of the standard power and actual power absorbed by the pump during oil transfers with pressure at 2 °C; (**c**) Variation curve of the mechanical efficiency absorbed by the pump during oil transfers with pressure at 25 °C; (**d**) Variation curve of the mechanical efficiency absorbed by the pump during oil transfers with pressure at 2 °C.

Furthermore, Fig. 9 illustrates the comparative measurement of the theoretical power, actual efficiency, and mechanical efficiency changes under various pressures when the buoyancy-driven system is operated at 2 °C and 25 °C. The standard power, actual power, and mechanical efficiency of the buoyancy-driven system tended to increase with increasing pressure, and the maximum power consumption of entire system did not exceed 115 W.

### Pressure testing of the full FUXING prototypes

**Figure 10:**
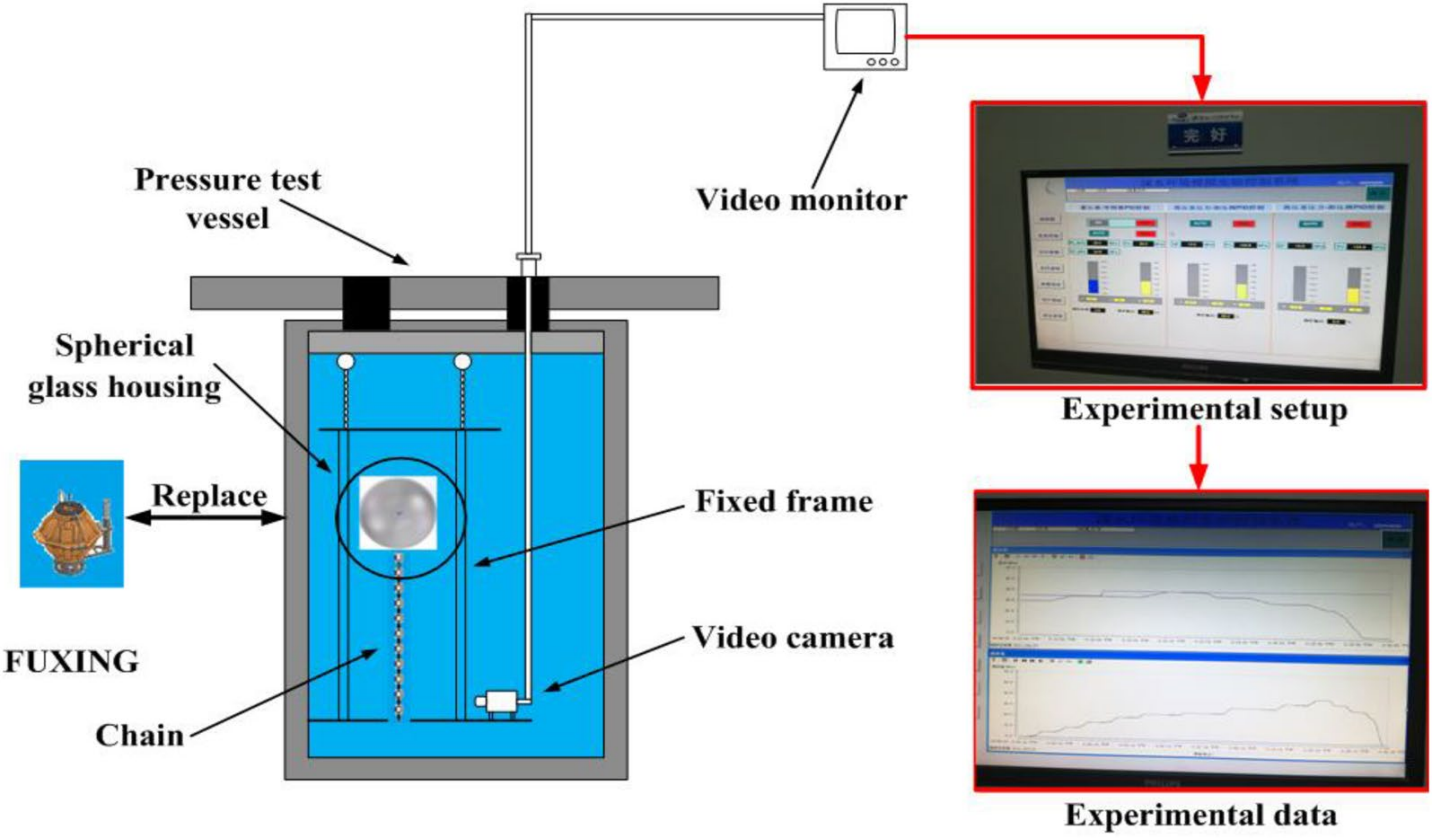
Schematic of the pressure testing vessel system: (The red box represents the experimental pressure setup and data display of the pressure testing vessel system).

Pressure testing is a critical part of preparation, and a key to success in sea experiments. The purpose of the test is to check the ability of the FUXING to withstand the maximum operating pressure in the pressure testing vessel. A schematic of the pressure testing vessel system is in Fig. 10. The experimental system of the pressure testing vessel includes a fixed frame, video camera, chain, FUXING, and video monitoring, which were placed inside the pressure testing vessel. The video monitor was placed outside the pressure-testing vessel. FUXING was placed inside the fixed frame, and the chain is connected to its bottom. The fixed frame not only protects FUXING, but also serves as a platform for the connection of the video camera. FUXING is equipped with a CTD sensor. The temperature and pressure were monitored by CTD sensors in the pressure-testing vessel. Two different tests were performed in the pressure-testing vessel system. The first was aimed at the compression of FUXING hull at a predefined depth. The purpose of the second test was to check the ability of FUXING to withstand maximum operating pressure.

A method is utilized to analyze the compression of FUXING hull at a given pressure, which lifts a chain in the pressure-testing vessel. The hull compression on FUXING with or without protective shells was simulated using a pressure testing vessel under the actual environmental conditions of deployment. The pressure testing vessel was filled with normal tap water in the experiment, and S = 0. The normal tap water temperature of the pressure-testing vessel system was measured to 25.425 °C. The normal tap water temperature remained constant during the experimental process. The effect of water temperature on the change in FUXING volume can be ignored. The water temperature was approximated as a constant. The change in the water density caused by the increasing external seawater pressure was only considered in this study. The precision of the temperature and pressure measurements was 0.003 °C and 0.04 MPa, respectively. The chain was composed of 37 weights. Each weight was 10 g. The total weight of the chain was 370 g, and its approximate volume was 32.7 ml at 1 atm. This is because the buoyancy of the chain is caused by the variation in the chain volume during pressurization. Its effect on the buoyancy change of FUXING in the water cannot be ignored during pressurization. The actual value of the lifted chain mass is the difference between the experimental and the buoyancy values of the lifted chain in this study.

**Figure 11:**
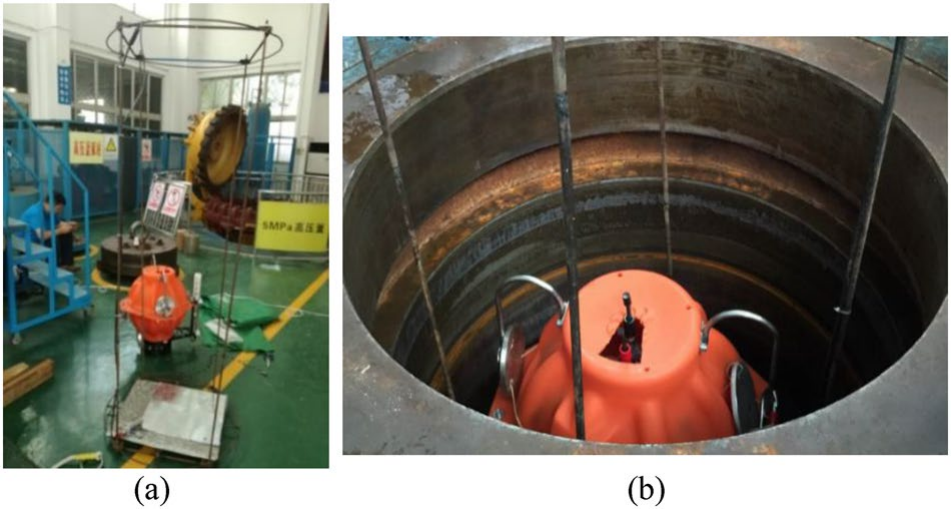
Operational mission in the pressure test vessel system: (**a**) experimental preparation process; (**b**) sealing process of the pressure test vessel system.

At the beginning of the pressure testing experiment, the pressure-testing vessel is filled with normal tap water. FUXING with protective shells is rested on the base of the fixed frame inside the pressure test vessel, which is then sealed off. (refer to Fig. 11).The maximum test static pressure of the “maximum diving depth” is set to a pressure equivalent to 4000 m. Pressure is added with a pressure incremental step of 2 MPa. When the pressure reaches the preset pressure value, the dwell time is 5 min. This is because the compression extent of the FUXING volume caused by the increased pressure is less than that of the surrounding water during pressurization. FUXING begins to ascend when its gravity is less than the buoyancy. The excessive buoyancy lifts the chain attached to the bottom of the FUXING. According to reference^29,30^, the compression of FUXING with protective shells is calculated by the weight of the lifted chain under the.

Subsequently, the pressure gradually decreases. After the experiment, the FUXING with protective shells is removed, and the pressure hull confirmed that there is no seal leak or abnormal deformation. Pressure testing with the measured CTD data acquisition can assure the actual results of the normal tap water density as intended. The hydrostatic proof test of FUXING with protective shells is validated against the effects of external pressure. Protective shells of FUXING are removed. The chain is connected to the bottom of the spherical glass housing. The spherical glass housing is placed in the water of the pressure test vessel. The pressure testing of the spherical glass housing is s repeated according to the above experimental procedure. The pressure incremental step and holding time at pressure during pressurization are the same as in the above experimental process. Similarly, the compression of FUXING without protective shells is calculated by the weight of the lifted chain under the corresponding pressure during pressurization.

**Figure 12:**
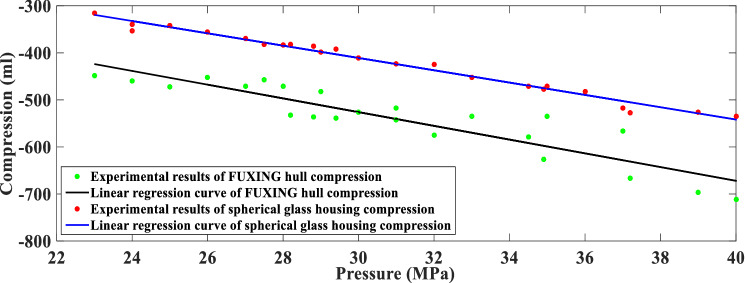
Compression linear regression curve of the FUXING hull and spherical glass housing under various pressure levels.

**Figure 13:**
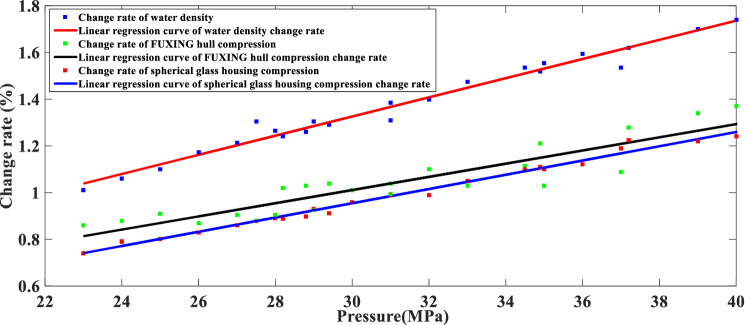
Linear regression curve of the change rate in the water density and FUXING hull compression under various pressure levels.

Pressure testing makes sense to obtain the compression of FUXING hull at various external pressures. To compare the compression between FUXING with and without protective shells, the compression experimental results of FUXING shell and spherical glass housing are obtained by the pressure test vessel system, as illustrated in Fig. 12. It can be determined that the ability of FUXING is checked to withstand a maximum operating pressure of 40 MPa. In addition, the experimental results indicate that the compression of FUXING shell is mainly determined by the compression of the spherical glass housing. To study the difference in compressibility between the water and the FUXING hull, the linear regression curve of the change rate in the water density and the FUXING hull compression with pressure is compared in Fig. 13. It can be observed that under a pressure of 40 MPa compared to 23 MPa, the increase rate of water density is 0.715%, the compression rate of the FUXING shell is 0.51%, and the compression rate of the spherical glass housing was 0.49%. Thus, the compressibility of FUXING with protective shells is closer to that of water than that of FUXING without protective shells. If only the pressure effect is considered, when the compressibility of FUXING approximately coincides with water, FUXING will achieve a state of neutral buoyancy at any specified depth. The compressibility and sealability of FUXING hull met the requirements of the maximum diving depth.

## At-sea experimental results

**Figure 14:**
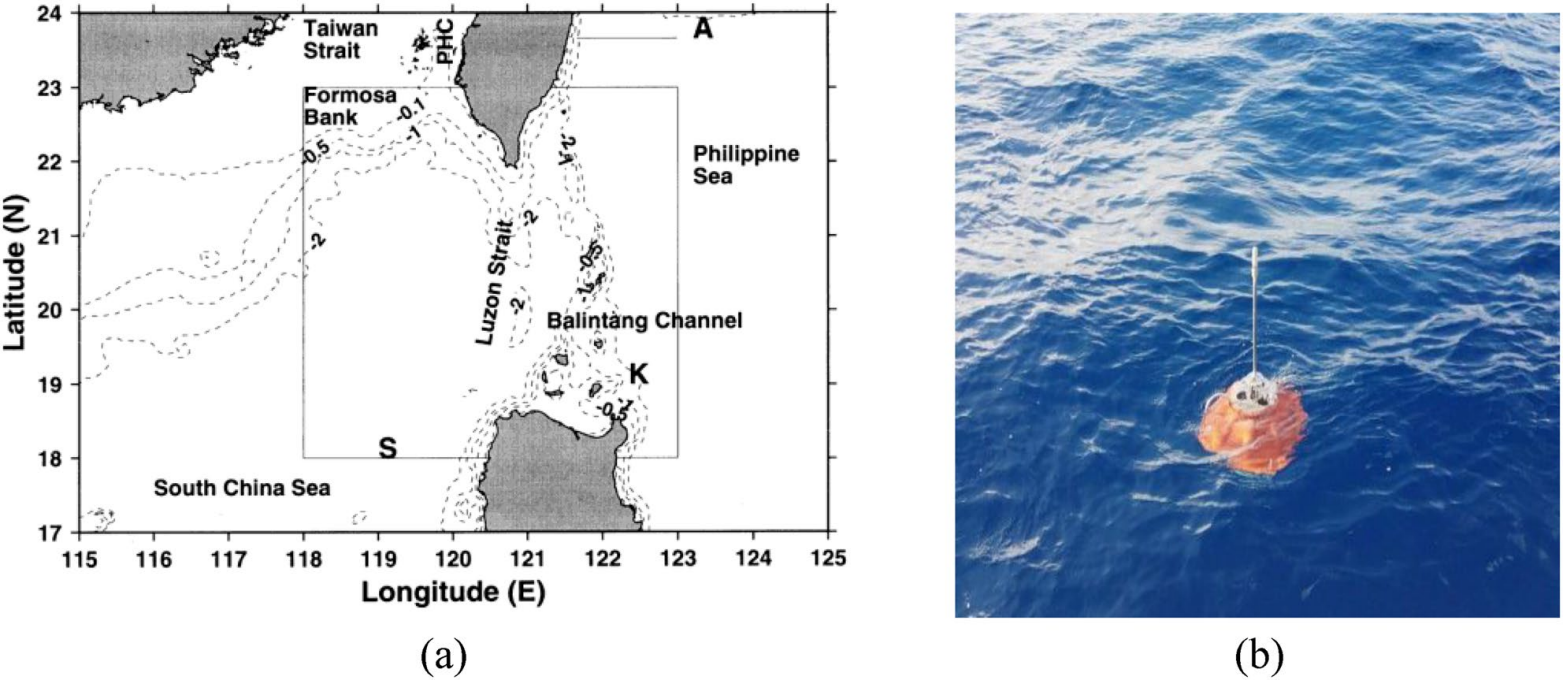
At-sea experiments of the FUXING: (**a**) Position of the FUXING deployed in the Pacific Ocean, northeast off the Luzon Island, Philippines (19.08° N, 124.66° E); (**b**) The complete prototype system of FUXING.

**Figure 15:**
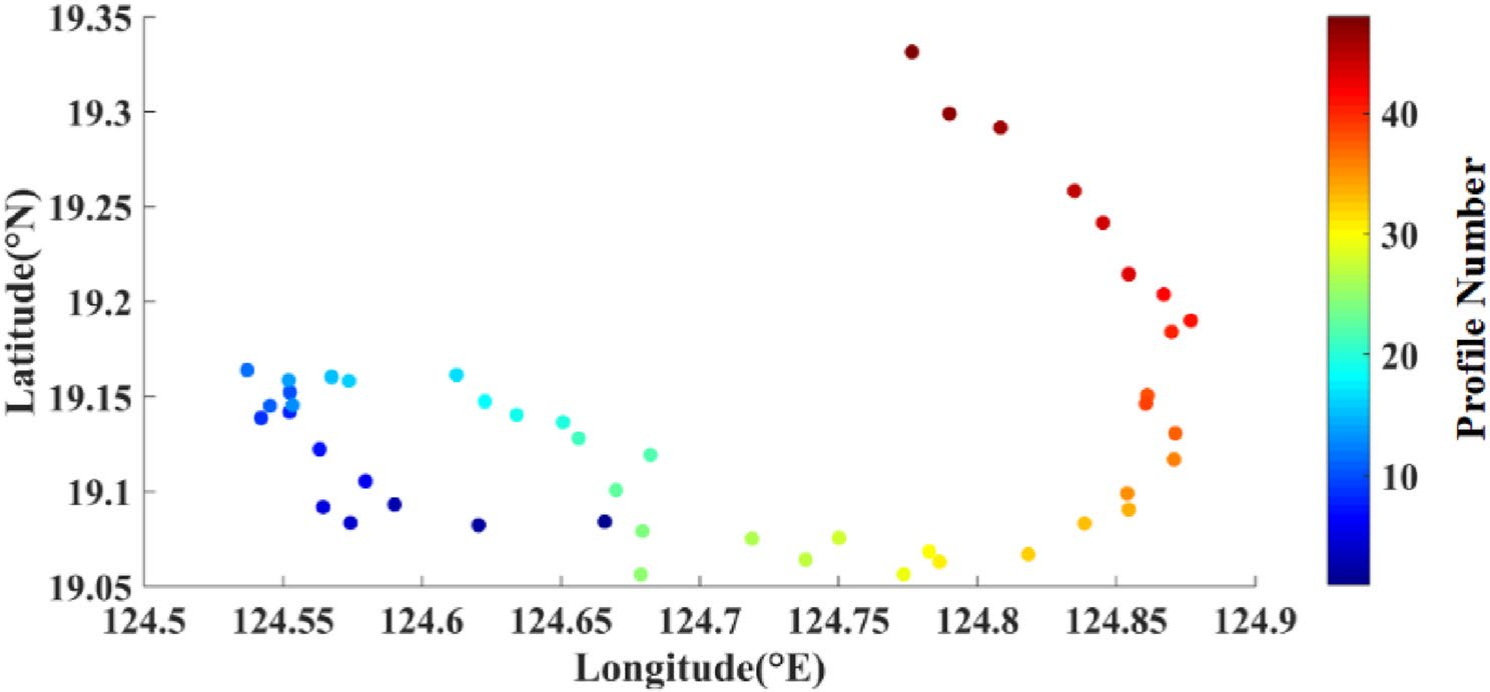
Drift track of the FUXING deployed in the Pacific Ocean, northeast off the Luzon Island, Philippines (19.08° N, 124.66° E).

A complete prototype system of FUXING was successful at sea exposure. FUXING was deployed in the Pacific Ocean, northeast off the Luzon Island, Philippines (19.08° N, 124.66° E) in August 2020, returning scientific data from depths of over 4041 m. The FUXING was equipped with a CTD sensor (Sea-Bird Electronics, Inc., SBE61) to measure the depth, conductivity and temperature. Relevant parameters of the CTD sensor are shown in Table 5. FUXING was remotely controlled and programmed cyclically to incrementally increase its depth. Pre-programmed missions were performed to collect profiling time-series data, and to test the reliability and overall float performance for longer missions. After 48 cycles, the batteries were almost flat. A drift track from an initial test with FUXING was deployed in a dynamic area of the the Pacific Ocean, northeast off the Luzon Island, Philippines (19.08° N,124.66° E) (see Fig. 14). The trajectory of the FUXING with the deployment site was marked by a dot (refer to Fig. 15). The FUXING drifted in a southwest direction, and then turned south.

**Table 5:** Relevant performance parameters of the CTD sensor (Sea-Bird Electronics, Inc., SBE61).

	Measurement range	Initial accuracy	Resolution
Temperature	− 5~35 °C	0.002 °C	0.0001 °C
Salinity	0~9 S/m	0.0003 S/m	0.00005 S/m
Depth	0~10500 m	0.1%FS	0.002%FS

**Figure 16:**
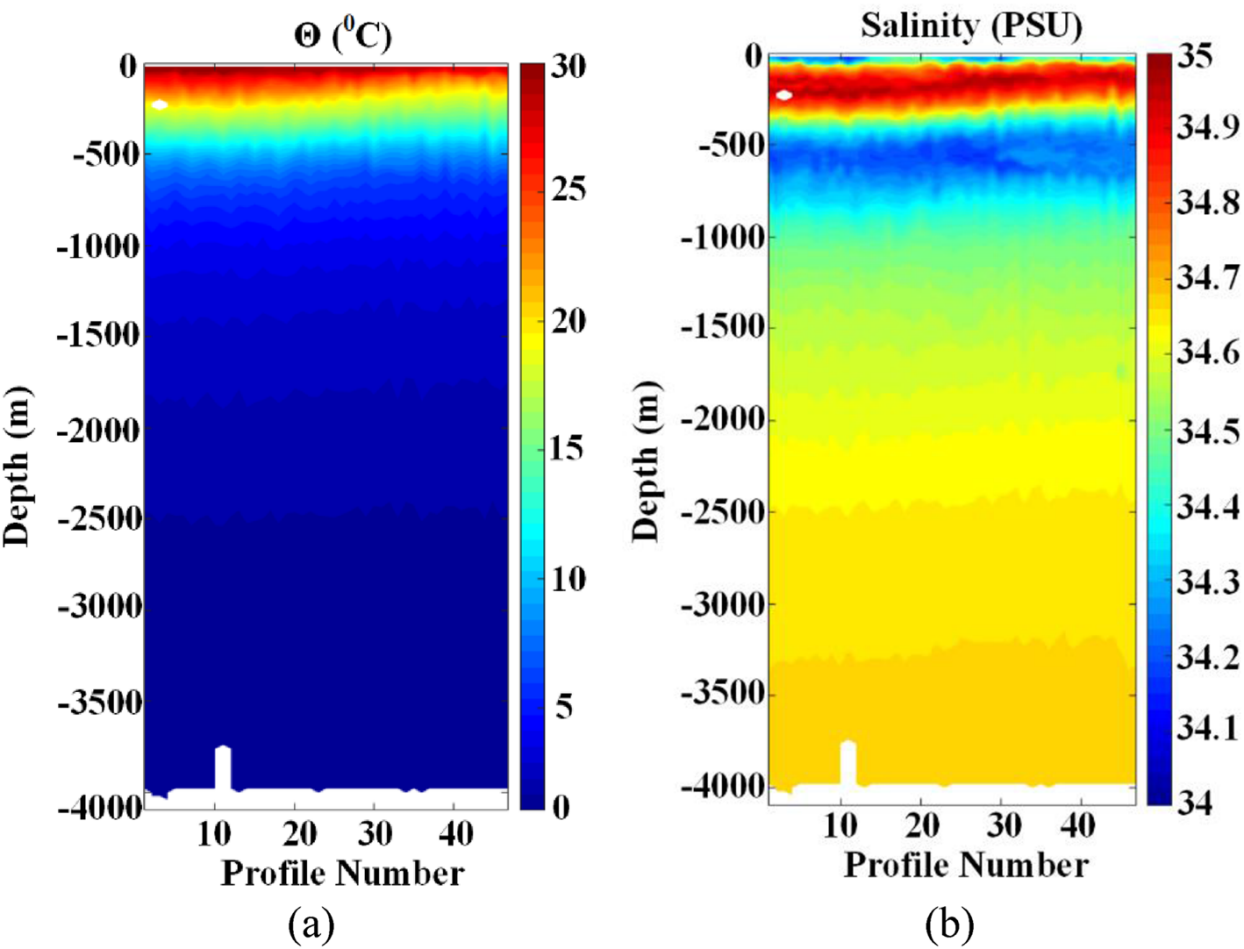
Seawater temperature, salinity and depth data from sea trials: (**a**) Relationship between seawater temperature and seawater depth; (**b**) Relationship between seawater salinity and seawater depth.

FUXING dove to over 4041 m for the first time in the Pacific Ocean, northeast off the Luzon Island, Philippines. This was the first step in our technological development. Figure 16 illustrates the maximum depth and profile number obtained by FUXING during tests in August 2020, with pressure measurements starting at approximately 4041 dbar. The reproducibility of FUXING behavior mainly includes the regularity of the temporal cycles, the stability of the FUXING at parking depth, and the quality of data transmission. The regularity of the temporal cycles and the stability of FUXING at parking depth indicated good reproducibility. The ascending profile was performed at a speed of 0.09 m/s with a standard deviation of 0.005 m/s. In addition, the assessment of the designed buoyancy-driven system proved that the objective of 48 cycles at 4000 m was realistic. The drift at the parking depth interval ($\pm$ 41 m) was particularly stable. In order to verify the effectiveness of the optimization of the oil draining adjustment mode, the total energy consumption of the profile motion was collected before and after the optimization. Before the optimization of the energy consumption in the floating stage, the trial energy consumption showed that the float requires around 101.7 kJ for a 4000 m profile observation. Using the oil draining adjustment mode in the floating process, a total energy consumption of around 78.8 kJ was achieved for a typical 4000 m profile.

Approximately 12 buoyancy-driven system actions were needed between 4000 m and the surface. The temperature and salinity profiles collected by FUXING (displayed in Fig. 16) indicated an overall good quality of the transmitted data. The good quality and stability of the satellite telemetry performances were verified. It can be observed that the temperature distribution tended to decrease from the surface layer (> 20 °C) to the deep layer (< 3 °C), the upper layer isotherms were densely distributed, and the lower layer was sparse. The densest isotherms occurred at a depth of 100–500 m, and the isotherms were undulating. The temperature gradually decreased from 30 °C in the surface layer to approximately 10 °C in the vicinity of the 500 m layer, and the temperature below 500 m layer continued to decrease. It can be observed that the salinity observed by FUXING indicated a two-low and one-high distribution trend from the surface to the bottom. The first relatively low salt zone (approximately 34.40 PSU on average) appeared in the surface layer, while the second (34.15–34.30 PSU) was approximately the 700 m layer. The relatively high-salt area was near the water depth of 300 m, and its value was between 34.80 PSU and 35.10 PSU.

**Figure 17:**
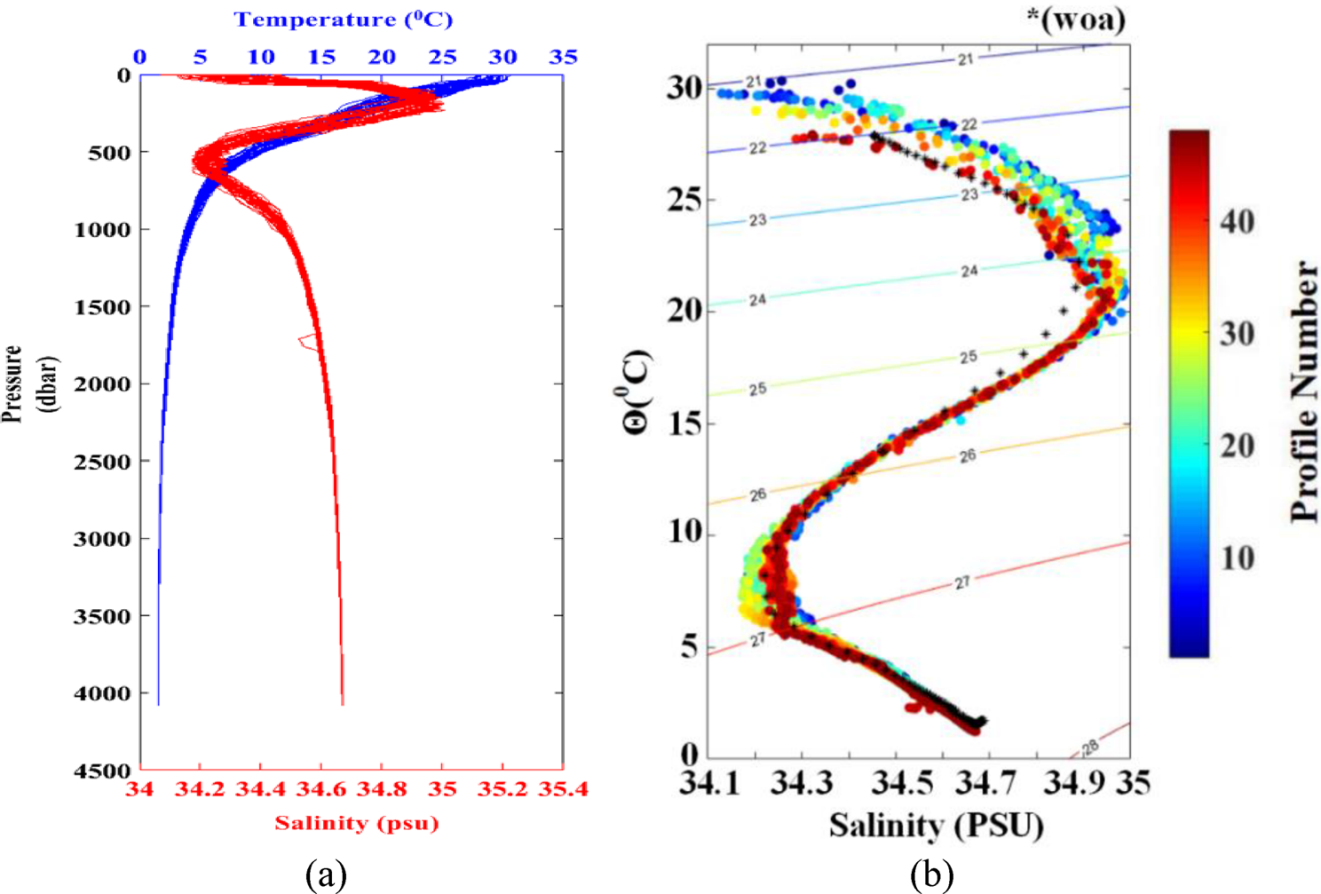
Distribution of seawater temperature and salinity in sea trials: (**a**) measured pressure versus temperature & salinity from 4000 m; (**b**) θ-S point cluster diagram of the FUXING.

The temperature-salinity distribution curve of the FUXING (refer to Fig. 17) illustrated a "horse tail" shape, that is, the top was thick and the bottom was thin. In Fig. 17, the subsurface high temperature and high salinity inflection point temperature is about 21 °C, and the salinity is lower than 35 PSU; while the latter inflection point temperature is about 25 °C, and the salinity is higher than 34.9 PSU. In addition, the inflection point temperature of the deep low temperature sub-high brine is about 7 °C, and the salinity is higher than 34.1 PSU.

**Figure 18:**
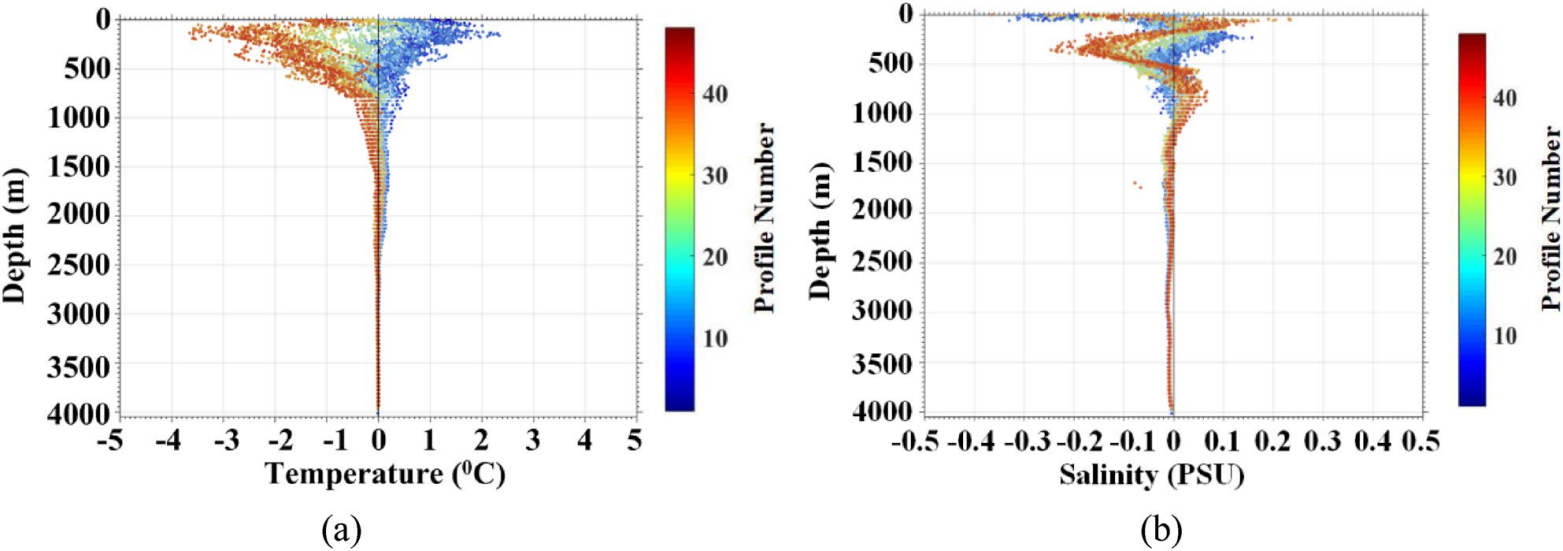
Error analysis of seawater temperature and salinity in sea trials: (**a**) error analysis of seawater temperature; (**b**) error analysis of sea water salinity.

As illustrated in Fig. 18, the collected temperature data were consistent with the historical data of WOA2018^31,32^. The collected salinity data by FUXING was approximately a salinity bias of 0.008~0.009PSU. The salinity accuracy requirements of 0.01PSU was fully met by the international Argo plan. As with the vertical distribution of temperature, the upper salinity curve was more dispersed, and the salinity changed less as the FUXING deepened. Tests at sea are still ongoing. The reproducibility of FUXING behavior needs to be tested.

## Discussion

In order to improve the ability of the profile observation in the deep ocean below 2000 m, a profile buoy named FUXING was studied, and the buoyancy-driven system was designed. The current buoyancy-driven system has problems such as air lock, high energy consumption and failure to achieve accurate buoyancy regulation. To make the designed buoyancy-driven system complete the accurate buoyancy adjustment, a draw-wire displacement sensor was adopted to measure the oil quantity of the internal reservoir to realize the precise buoyancy regulation. The combination of the high-pressure plunger pump and air pump was adopted for the designed buoyancy-driven system, and was placed in the spherical glass housing under the negative pressure. In order to solve the problem of air lock, the air pump was used to supplement air pressure to the internal oil reservoir at regular intervals to improve the oil pumping capacity of the high-pressure plunger pump to the external oil bladder. The calculation model of the pressure change inside the spherical glass housing when it is submerged and floated was given. Considering the temperature difference between the water depth of 4000 m and the sea surface, when the minimum oil absorption pressure of the high-pressure piston pump is 0.8 bar, the internal gas pressure of the spherical glass housing is 0.517 bar when it floats. When the maximum oil absorption pressure of the high-pressure piston pump is 1.5 bar, the internal gas pressure of the spherical glass housing is 0.488 bar when it floats. In order to reduce the energy consumption of FUXING and extend its service life, by introducing the external seawater pressure as the driving force, the low power consumption diving process of FUXING was effectively solved. The oil draining optimization adjustment mode the FUXING’s floating process was established by combining the dynamic model of FUXING. The reliability test of the buoyancy-driven system showed that when the frequency of the oil draining was one time and 15 times, respectively, the total energy consumption of FUXING was reduced from 96.4 to 73 kJ. The total energy consumption saving was about 24.2%. The tests at sea of FUXING revealed that the total energy consumption for one-time oil draining was 101.7 kJ, compared with 78.8 kJ for a 15 times oil draining. The optimized oil draining adjustment mode saves 22.5% of the total energy consumption. The optimization effect of the oil draining adjustment mode was verified.

In order to study the mixing characteristics of the surface water masses in the northeast off the Luzon Island, the collected temperature, salinity and depth data in the sea trials were effectively analyzed. The profile data from sea trials showed that the water structure in the northeast off the Luzon Island area was characterized by the high temperature and low salinity in the surface layer, high temperature and high salinity in the subsurface layer, low temperature and low salinity in the middle layer, and low temperature and sub-high salinity in the deep layer. The dispersion of the temperature and salt point aggregation in the surface, subsurface and middle water bodies reflected the mixing intensity of water bodies in this layer. In the θ-S point cluster diagram, all the dots (salinity maximum data) ranged between 34.1PSU and 35PSU. Because the θ-S point cluster values of the anomalously high-salinity water were between the mean θ-S point cluster values in the northern South China Sea and north Pacific subsurface water^33^. The results as mentioned above showed that the outer sea water gradually mixes with the South China Sea water after passing through the northeast off the Luzon Island, implying that the subsurface waters share characteristics with the North Pacific Tropical Water. Therefore, it is of scientific and practical value to investigate the changes in the Kuroshio intrusion and the hydrological cycle characteristics of the Tropical Water.

## Conclusion

To extend the deep ocean observation depth of 2000 m down to 4000 m, this study presented a novel buoyancy-driven self-sustaining profile float that had been applied to ocean observations in the northeast off the Luzon Island. For the buoyancy-driven system, it was proposed to utilize a draw-wire displacement sensor to accurately measure the displacement of the piston in the inner oil reservoir to realize the precise buoyancy adjustment of FUXING. Simultaneously, an air pump was utilized to provide an appropriate pressure for the internal oil reservoir to solve the air lock problem in the system. To ensure the oil pumping capacity of the high-pressure plunger pump, the variation in gas pressure inside the spherical glass housing under the maximum and minimum oil suction pressures of the high-pressure piston pump were discussed. To reduce the energy consumption of the buoy operation process, the optimized oil draining adjustment mode in the floating process was proposed. It is found that the total energy consumption of FUXING is nonlinear with the oil draining frequency. When the floating process is adjusted 15 times for a buoyancy, the energy consumption is the lowest. A reliable buoyancy adjustment cycle was completed for the designed buoyancy-driven system under various pressures of 0–40 MPa at 2 °C or 25 °C in the hydraulic engine characterization test. The anti-pressure performance of the designed FUXING was checked by a pressure resistance test to withstand the maximum operating pressure of 40 MPa. Longer missions were planned to be conducted in the northeast off the Luzon Island in August 2020, where the overall FUXING performance would be initially tested. The results of the at-sea experiments indicated that the CTD sensor carried by the designed FUXING in the sea trials realized a maximum working depth of 4041 m and the requirement for continuous measurement of 48 profiles. The obtained temperature, salinity, and depth data had an overall good quality of hydrological data acquisition and transmission, thus, verifying the stability of the FUXING to carry multiple sensors and continuous hydrology measurement of multiple profiles. The results showed that the optimized oil draining adjustment mode can be used as the profiling float’s buoyancy regulation strategy to optimize the energy consumption of FUXING. The mixing characteristics of the external sea water mass passing through the northeast sea area of Luzon Island are effectively observed by the collected hydrological data. It is found that the outer sea water gradually mixes with the South China Sea water after passing through the northeast off the Luzon Island. The characteristics and distribution of the Tropical Water near the northeast off the Luzon Island area will be further analyzed.

## References

[1] Smith RN, Huynh VT (2014). Controlling buoyancy-driven profiling floats for applications in ocean observation. IEEE J. Ocean. Eng..

[2] Liu G, Chen G, Jiao J (2015). Dynamics modeling and control simulation of an autonomous underwater vehicle. J. Coast. Res..

[3] D'Asaro EA (2003). Performance of autonomous Lagrangian floats. J. Atmos. Ocean. Technol..

[4] Bishop JK, Davis RE, Sherman JT (2002). Robotic observations of dust-storm enhancement of carbon biomass in the north Pacific. Science.

[5] Nathalie, Z. & Guillaume, M. Report on the deep Argo implementation workshop. *Deep Argo Implementation Workshop*. Hobart, May 5–7th, 2015.

[6] Swallow JC (1953). A neutral-buoy float for measuring deep current. Deep Sea Res..

[7] Rossby T, Webb D (1970). Observing abyssal motion by tracking Swallow floats in the SOFAR channel. Deep Sea Res..

[8] Rossby T, Dorson D, Fontaine J (1986). The RAFOS system. J. Atmos. Ocean. Technol..

[9] Swift DD, Riser SC (2009). RAFOS floats: Defining and targeting surfaces of neutral buoyancy. J. Atmos. Ocean. Technol..

[10] Davis RE, Webb DC, Regier LA, Dufour J (1992). The autonomous Lagrangian circulation explorer (ALACE). J. Atmos. Ocean. Technol..

[11] Davis RE, Sherman JT, Dufour J (2001). Profiling ALACEs and other advances in autonomous subsurface floats. J. Atmos. Ocean. Technol..

[12] Gould WJ (2005). From swallow floats to Argo—the development of neutrally buoyant floats. Deep Sea Res. Part II.

[13] Hanling W, Yanjun L, Gang X (2018). Design and experimental research on deep water pressure resistance system of Argo float. J. Coast. Res..

[14] Izawa, K., *et al*. On the weight adjustment of profiling floats. ARGO Technical Report FY2001, Japan Marine Science and Technology Center, 18–35 (2002).

[15] Kobayashi, T., *et al*. Deep NINJA: A new profiling float for deep ocean observation. In *The Twenty-second International Offshore and Polar Engineering Conference*, 454–461 (OnePetro, 2012).

[16] Le Reste S, Dutreuil V, André X (2016). “Deep-Arvor”: A new profiling float to extend the Argo observations down to 4000-m depth. J. Atmos. Ocean. Technol..

[17] Petzrick, E., Truman, J. & Fargher, H. Profiling from 6,000 meter with the APEX-Deep float. In *2013 OCEANS-San Diego*, 1–3 (IEEE, 2013).

[18] Roemmich D, Sherman JT, Davis RE (2019). Deep SOLO: A full-depth profiling float for the Argo Program. J. Atmos. Ocean. Technol..

[19] Wang Q, Qiu Z, Li H, Yang S, Li X (2019). Ballasting weight on net buoyancy changes and submergence depth for a spherical buoyancy–driven intelligent float based on the ballasting method. IEEE Access.

[20] Qiu Z, Wang Q, Li H, Yang S, Li X (2019). Depth control for a deep-sea self-holding intelligent buoy under ocean current disturbances based on finite-time boundedness method. IEEE ACCESS.

[21] Kobayashi, T., *et al*. Deep NINJA: A new float for deep ocean observation developed in Japan. In *2011 IEEE Symposium on Underwater Technology and Workshop on Scientific Use of Submarine Cables and Related Technologies*, 1–6 (2011).

[22] Collins CA, Margolina T, Rago TA (2013). Looping RAFOS floats in the California current system. Deep Sea Res. Part II.

[23] D’Asaro EA, Farmer DM, Osse JT (1996). A Lagrangian float. J. Atmos. Ocean. Technol..

[24] Mu W, Zou Z, Liu G (2019). Depth control method of profiling float based on an improved double PD controller. IEEE Access.

[25] Bai Y, Hu R, Bi Y (2022). Design and depth control of a buoyancy-driven profiling float. Sensors.

[26] Elkolali M, Al-Tawil A, Alcocer A (2022). Design and testing of a miniature variable buoyancy system for underwater vehicles. IEEE Access.

[27] Veeraragavan S, Maurya S, Suresh G, et al. Optimization of Deep-sea Profiling Float based on Ballasting Methodology. OCEANS 2022-Chennai. IEEE, 2022, 1–5.

[28] Liu Y, Guo F, Zhai X (2020). Research on the operational stability and energy consumption of the profiling float. J. Coast. Res..

[29] Pausch S, Below D, Hardy K (2009). Under high pressure: Spherical glass flotation and instrument housings in deep ocean research. Mar. Technol. Soc. J..

[30] Reynolds T, Lomacky O, Krenzke M (1973). Design and analysis of small submersible pressure hulls. Comput. Struct..

[31] Shen H, Li L, Li J (2021). The seasonal variation of the anomalously high salinity at subsurface salinity maximum in northern south china sea from Argo data. J. Mar. Sci. Eng..

[32] Yu L (2021). Revisiting the global patterns of seasonal cycle in sea surface salinity. J. Geophys. Res. Oceans.

[33] Sun Q, Little CM, Barthel AM (2021). A clustering-based approach to ocean model–data comparison around Antarctica. Ocean Sci..

